# TLR11-independent inflammasome activation is critical for CD4+ T cell-derived IFN-γ production and host resistance to *Toxoplasma gondii*

**DOI:** 10.1371/journal.ppat.1007872

**Published:** 2019-06-13

**Authors:** Américo H. López-Yglesias, Ellie Camanzo, Andrew T. Martin, Alessandra M. Araujo, Felix Yarovinsky

**Affiliations:** Center for Vaccine Biology and Immunology, University of Rochester, Rochester, NY United States of America; University of California Davis School of Veterinary Medicine, UNITED STATES

## Abstract

Innate recognition of invading intracellular pathogens is essential for regulating robust and rapid CD4+ T cell effector function, which is critical for host-mediated immunity. The intracellular apicomplexan parasite, *Toxoplasma gondii*, is capable of infecting almost any nucleated cell of warm-blooded animals, including humans, and establishing tissue cysts that persist throughout the lifetime of the host. Recognition of *T*. *gondii* by TLRs is essential for robust IL-12 and IFN-γ production, two major cytokines involved in host resistance to the parasite. In the murine model of infection, robust IL-12 and IFN-γ production have been largely attributed to *T*. *gondii* profilin recognition by the TLR11 and TLR12 heterodimer complex, resulting in Myd88-dependent IL-12 production. However, TLR11 or TLR12 deficiency failed to recapitulate the acute susceptibility to *T*. *gondii* infection seen in *Myd88*^-/-^ mice. *T*. *gondii* triggers inflammasome activation in a caspase-1-dependent manner resulting in cytokine release; however, it remains undetermined if parasite-mediated inflammasome activation impacts IFN-γ production and host resistance to the parasite. Using mice which lack different inflammasome components, we observed that the inflammasome played a limited role in host resistance when TLR11 remained functional. Strikingly, in the absence of TLR11, caspase-1 and -11 played a significant role for robust CD4+ T_H_1-derived IFN-γ responses and host survival. Moreover, we demonstrated that in the absence of TLR11, production of the caspase-1-dependent cytokine IL-18 was sufficient and necessary for CD4+ T cell-derived IFN-γ responses. Mechanistically, we established that *T*. *gondii*-mediated activation of the inflammasome and IL-18 were critical to maintain robust CD4+ T_H_1 IFN-γ responses during parasite infection in the absence of TLR11.

## Author summary

It is currently estimated that one third of the world’s population is seropositive for the parasite *Toxoplasma gondii* and this parasite can lead to serious illness and death in immunocompromised patients, and is one of the leading causes of foodborne-related deaths in the United States. Host immunity against the parasite has largely been attributed to recognition of the parasite-derived protein, profilin, by the innate Toll-like receptors (TLRs), TLR11 and TLR12. *T*. *gondii* also triggers inflammasome activation in a caspase-1-dependent manner resulting in cytokine release. However, how these innate recognition systems regulate T_H_1 immunity and host resistance remains largely unknown. Therefore, using genetically modified mice, we investigated TLR11-dependent and -independent host immunity against the parasite. Our research establishes that in the absence of TLR11, inflammasome activation and subsequent production of the inflammasome-dependent molecule, IL-18 are critical for host immunity to the parasite. These data provide novel mechanistic insight into how TLR and inflammasomes cooperate in regulation of T_H_1 immunity and host protection.

## Introduction

*Toxoplasma gondii* is an obligate intracellular apicomplexan parasite capable of infecting most nucleated cells of warm-blooded animals, including humans, resulting in persistent cysts residing within the skeletal muscle, cardiac tissue, and brain [1–3]. Notably, in the United States toxoplasmosis is a leading cause of foodborne-related deaths [4, 5]. A detailed understanding of the cellular and molecular mechanisms responsible for host resistance against *T*. *gondii* has been well-established in murine infection models [6]. Parasite recognition by TLRs is critical for IL-12 production by dendritic cells (DCs), which is critical for a rapid and robust CD4+ T helper 1 (T_H_1) response leading to production of IFN-γ [7–10].

During *T*. *gondii* infection both IL-12 and IFN-γ cytokines are essential for host resistance to *T*. *gondii*. The cytokine IFN-γ is critical for macrophage activation and induction of IFN-γ-inducible genes including p47 immunity related GTPases (IRGs) and p65 guanylate-binding proteins (GBPs), which are required for intracellular parasite clearance [11–15]. Robust IL-12 production during mouse infection is largely attributed to direct recognition of *T*. *gondii* profilin by TLR11 which forms a heterodimer complex with TLR12 and leads to the activation of Myd88-dependent signaling pathways [16–21]. Unlike in mice, humans lack both these innate parasite ligand receptors; however, in most cases, humans that become infected with *T*. *gondii* are relatively resistant to this pathogen, unless patients become immunocompromised in CD4+ T cell effector functions [22, 23]. At present, TLR11-independent mechanisms of *T*. *gondii* recognition required for establishing T cell immunity are largely unknown; however, several recent studies including our own have suggested that CCL2-mediated recruitment of monocytes and the inflammasome-dependent release of mature IL-1β and IL-18 represent the initial steps in driving a TLR11-independent protective immunity to the parasite [24–28].

Activation of inflammasome sensors results in recruitment of the adaptor molecule apoptosis-associated speck-like protein containing a CARD (ASC) that is essential for activating caspase-1 and for processing IL-1β and IL-18 into biologically active forms [29–31]. Data from several groups have identified that both the NLRP1 and NLRP3 inflammasomes play a role in caspase-1-dependent release of IL-1β in response to *T*. *gondii* invasion *in vitro* [26, 28, 32]. Additional studies have shown that NLRP3, ASC, and IL-18 play a role in host resistance against *T*. *gondii in vivo* [32]; however, the precise *T*. *gondii*-derived stimuli that can initiate inflammasome activation remain undetermined.

In this report, we set out to determine the mechanisms of TLR11-dependent and independent regulation of CD4+ T_H_1 response during *T*. *gondii* infection. Our data revealed that mice with individual deficiencies in TLR11, NLRP3, ASC, or caspase-1 and -11 (Casp1/11) did not increase host mortality and had a minimal impact on CD4+ T cell-derived IFN-γ production. Strikingly, combined deficiency in TLR11 and Casp1/11 resulted in rapid susceptibility to parasitic infection caused by impaired T cell-derived IFN-γ responses during infection.

Mechanistically, we revealed that that in the absence of TLR11, inflammasome activation and IL-18-mediate CD4+ T_H_1-derived IFN-γ responses to *T*. *gondii*. Correspondingly, in the absence of Casp1/11, TLR11-dependent IL-12 production is sufficient to generate robust T_H_1 responses *in vivo*. These results provided an explanation for the high susceptibility of *Myd88*-deficient mice, which is not observed in *T*. *gondii* infected *TLR11*- or *Casp1/11*-deficient mice. TLR11-dependent activation of Myd88 was sufficient for establishing T_H_1 immunity to the parasite due to large amounts of IL-12 necessary for T_H_1 polarization without inflammasome-dependent release of IL-18. Similarly, IL-18 and possibly other Casp1/11-dependent mediators were capable of driving T_H_1 immunity and provided a partial protection against the parasite.

## Results

### Inflammasome plays a limited role for host resistance against *T*. *gondii*

Inflammasome activation plays a major role in host defense against intracellular pathogens, including *T*. *gondii*, in part via inflammasome-dependent release of IL-1β and IL-18 [26, 32, 33]. Despite recent reports establishing that parasite infected cells can lead to the rapid activation of both the NLRP1 and NLRP3 inflammasomes [26, 28, 32, 34, 35], the precise molecular and cellular events responsible for this activation *in vivo* remains undefined.

**Fig 1:**
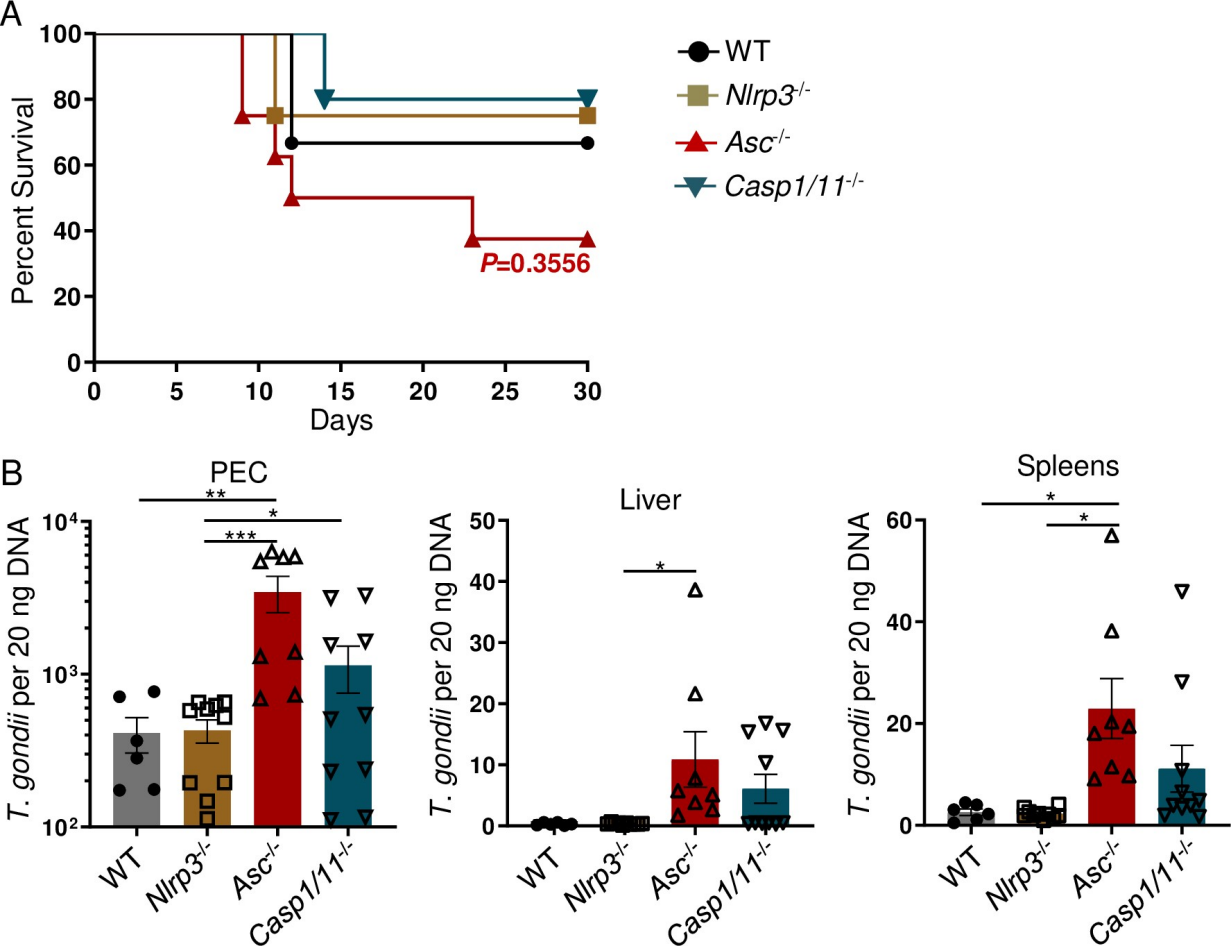
*The inflammasome plays a limited role in host resistance against* T. gondii. (**A**) Survival of WT, *Nlrp3*^-/-^, *Asc*^-/-^, and *Casp1/11*^-/-^ mice that were i.p. infected with 20 cysts of the ME49 strain of *T*. *gondii*. (**B**) Analysis of *T*. *gondii* parasite loads by qPCR in WT and inflammasome-deficient mice from PECs, livers, and spleens on day 8 of infection. Results are representative of three-independent experiments involving at least 3 mice per group. Statistical analyses of survival curve was done using Log-Rank (Mantel Cox) Test. All other statistical analyses were performed using one-way ANOVA with a Tukey’s multiple comparison test, *P<0.05, **P<0.01, ***P<0.001. Error bars, standard error mean.

To examine the role of the inflammasome in controlling host resistance to *T*. *gondii in vivo*, we assessed the susceptibility of mice lacking NLRP3 (*Nlrp3*^-/-^), ASC (*Asc*^-/-^), or *Casp1/11*^-/-^. We observed that upon intraperitoneal (i.p.) infection with cysts of the ME49 strain of *T*. *gondii*, mice deficient in the examined inflammasome components were relatively resistant to the acute stage of the infection (Fig 1A). Moreover, analysis of parasite burden on day 30 post-infection revealed that the examined inflammasome activation was not essential for the control of *T*. *gondii* during the persistent chronic stage of infection, as measured by cyst burden in the brains of infected mice (S1A Fig).

Inflammasomes play an important role during the initial encounter between host cells and invading pathogens; therefore, we examined if the inflammasome was required for local parasite restriction at the site of infection. Our results revealed that while NLRP3-deficiency had no effects on the parasite burden, mice lacking either ASC or Casp1/11 exhibited elevated local parasite loads compared to WT and *Nlrp3*^-/-^ mice (Fig 1B). As a consequence, the absence of ASC or Casp1/11, but not NLRP3, also resulted in elevated *T*. *gondii* dissemination during the acute stage of infection, as indicated by an increased pathogen burden in the liver and spleen of the infected mice (Fig 1B). These data revealed that, while the examined inflammasome components played a limited role in host survival during the acute stage of infection, both ASC and Casp1/11 participated in restriction of *T*. *gondii* tachyzoites during the acute stage of the infection. Nevertheless, the control of the parasite burden during the chronic stage of the infection in the brain did not require NLRP3, ASC, or Casp1/11.

### ASC, caspases-1 and -11 play a limited role for IFN-γ induction during *T*. *gondii* infection

The cytokine IFN-γ is essential for parasite clearance and host resistance [8], yet whether the inflammasome contributes to an IFN-γ response during parasite infection remains unclear. We first examined if IL-12, a key innate cytokine that drives IFN-γ production in NK, ILC1, and T_H_1 cells, was augmented by the inflammasome components NLRP3, ASC, or Casp1/11 in *T*. *gondii*-infected mice. We observed that deficiency in NLRP3, ASC, or Casp1/11 resulted in partially reduced circulating levels of IL-12 in the infected mice (S1B Fig). Similarly, *T*. *gondii* infected *Nlrp3*^-/-^, *Asc*^-/-^, and *Casp1/11*^-/-^ mice demonstrated modest reductions of IFN-γ within the sera on day 8 post-infection (S1C Fig). In agreement with the ELISA data, *IFN-γ* transcripts in the spleen were reduced in the absence of ASC and Casp1/11, further confirming a role for these inflammasome components in the regulation of immunity to *T*. *gondii* (S1D Fig). Next, we examined if local IFN-γ induction was also compromised. Unexpectedly, *IFN-γ* transcript levels locally at the site of infection in the peritoneum were not reduced in any of the infected inflammasome-deficient mice, and the elevated *IFN-γ* transcript levels in *Asc*^-/-^ mice was most likely caused by the increased pathogen burdens observed in those animals (S1D Fig and Fig 1B).

The cytokine IFN-γ plays a critical role in host defense by initiating a series of genes essential for parasite elimination and cell recruitment. Therefore, we examined if the absence of the inflammasome disrupted IFN-γ-mediated gene expression during *T*. *gondii* infection. In agreement with the largely unimpaired IFN-γ production at the site of infection, we observed robust induction of IFN-γ-inducible genes in both WT and inflammasome-deficient mice (S1E Fig). Similarly, lower levels of splenic *IFN-γ* transcripts in the absence of the inflammasome resulted in a reduction of splenic *Irgm3* and *Cxcl10* transcripts (S1F Fig). Thus, our data revealed a partial and limited role for ASC and Casp1/11 in the regulation of systemic IFN-γ responses during *T*. *gondii* infection. Unexpectedly, the examined inflammasome components were dispensable for the regulation of IFN-γ production and the expression of IFN-γ-induced genes at the site of infection.

### The inflammasome plays a limited role in parasite-mediated CD4+ T_H_1-derived IFN-γ responses

**Fig 2:**
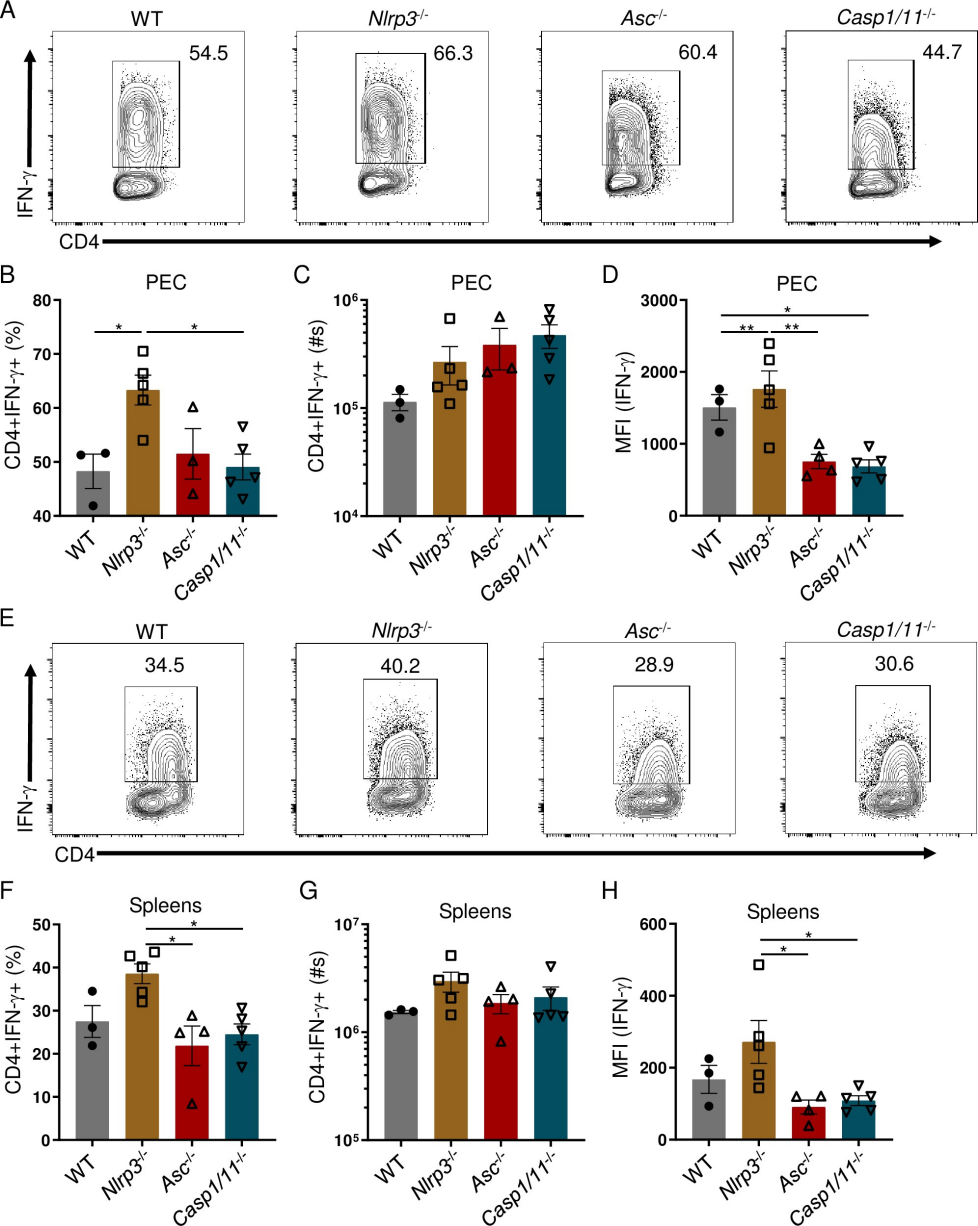
*The inflammasome has a limited role in stimulating* T. gondii-*mediated CD4+ T*_*H*_*1 derived IFN-γ responses*. (**A, E**) WT, *Nlrp3*^-/-^, *Asc*^-/-^, and *Casp1/11*^-/-^ mice were i.p. infected with 20 cysts of *T*. *gondii*. PECs (A) and spleens (E) were harvested and IFN-γ production by CD4+ T cells was analyzed by flow cytometry. (**B, C, F, G**) Average frequencies (B, F) and absolute quantification (C, G) of CD4+IFN-γ+ cells in the PECs and spleens were analyzed on day 8 following infection. (**D, H**) Mean fluorescent intensity (MFI) of CD4+ T cell IFN-γ in the PECs (D) and spleens (H) was analyzed on day 8 post-infection. Results are representative of three-independent experiments involving at least 3 mice per group. Statistical analyses were done using one-way ANOVA with a Tukey’s multiple comparison test, *P<0.05, **P<0.01. Error bars, standard error mean.

A potent T_H_1 response is a hallmark of *T*. *gondii* infection. It has been previously established that TLR-dependent production of IL-12 plays a major role in T_H_1 polarization, whereas IL-18 and IL-1β are able to enhance the robust IFN-γ production by CD4+ T cells [36–38]. However, it remains undetermined how the inflammasome impacts parasite-mediated T_H_1 effector function. Therefore, we assessed the contribution of the inflammasome in mediating the CD4+ T_H_1 response during *T*. *gondii* infection. We observed no reduction in the frequency or absolute cell numbers of peritoneal CD4+IFN-γ+ T cells in the absence of NLRP3, ASC, or Casp1/11 by day 8 post-infection (Fig 2A–2C). However, *Asc* and *Casp1/11* deficiency resulted in a reduction in the amount of IFN-γ produced by peritoneal CD4+ T_H_1 cells (Fig 2D). Similarly, we established that the examined inflammasome components NLRP3, ASC, and Casp1/11 played no discernible role in the induction of the systemic T_H_1 response analyzed in spleens of the infected mice (Fig 2E–2H); however, deficiency in NLRP3 resulted in exacerbated frequency and total cell numbers of splenic CD4+IFN-γ+ T cells, and the amounts of IFN-γ produced by splenic CD4+ Th1s as measured by intracellular staining for this cytokine compared to *Asc*^-/-^ and *Casp1/11*^-/-^ mice (Fig 2F–2H).

In addition to CD4+ T cells, several other cell types contribute to IFN-γ production in response to *T*. *gondii* infection, including CD8+ T cells, NK cells, and neutrophils. Our data reveals that the examined inflammasome components played no significant role in the regulation of IFN-γ responses by CD8+ T cells, NK1.1+ NK cells, or Ly6G+ neutrophils locally or in the spleen of the infected mice compared to WT controls (S2 Fig).

### TLR11-independent host resistance against *T*. *gondii* requires inflammasome activation

**Fig 3:**
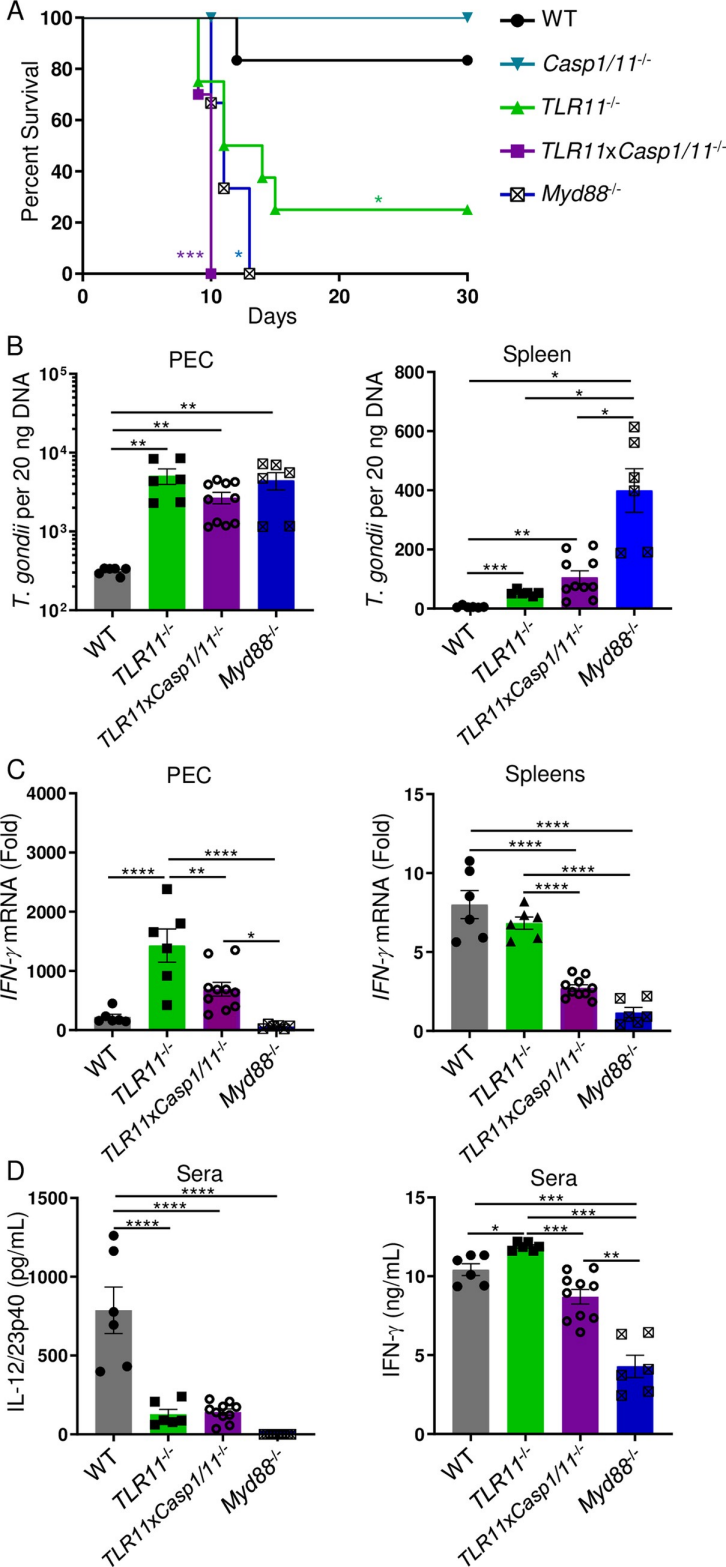
*The inflammasome is required for host resistance against* T. gondii *in the absence of TLR11*. (**A**) Survival of WT, *Casp1/11*^-/-^, *TLR11*^-/-^, *TLR11*x*Casp1/11*^-/-^, and *Myd88*^-/-^ (▼) mice that were infected i.p. with 20 cysts of ME49. (**B**) Analysis of *T*. *gondii* parasite loads by qPCR in WT, *TLR11*^-/-^, *TLR11*x*Casp1/11*^-/-^, and *Myd88*^-/-^ mice from PECs and spleens on day 8 of infection. (**C**) qRT-PCR analysis of relative *IFN-γ* expression measured in the PECs and spleens of mice infected with 20 cysts of *T*. *gondii* on day 8 post-infection. (**D**) Analysis by ELISA of serum IL-12/23p40 and IFN-γ in WT, *TLR11*^-/-^, *TLR11*x*Casp1/11*^-/-^, and *Myd88*^-/-^ mice infected with *T*. *gondii* was performed on day 8 of infection. Survival curve is a combination of two-independent experiments involving at least 3 mice per group. Statistical analyses of survival curve was done using Log-Rank (Mantel Cox) Test. All other results are representative of three-independent experiments involving at least 5 mice per group. Statistical analyses were done using one-way ANOVA with a Tukey’s multiple comparison test, *P<0.05, **P<0.01, ***P<0.001, ****P<0.0001. Error bars, standard error mean.

Direct innate recognition of *T*. *gondii* profilin by the TLR11/12 heterodimer complex, regulates a major Myd88-dependent mechanism of IL-12 mediated immunity against *T*. *gondii* [6]. Therefore, we hypothesized that an intact TLR11 signaling pathway masks a role for inflammasomes in regulating T_H_1 immunity towards *T*. *gondii* and host resistance to the parasite due to its dominant effect on the activation of innate immune cells [39]. To test this hypothesis, we examined a role for Casp1/11 in the absence of TLR11. Our results revealed that while neither TLR11 nor Casp1/11 alone were absolutely essential for host survival during acute toxoplasmosis, a combined deficiency in TLR11 and Casp1/11 (*TLR11*x*Casp1/11*^-/-^) resulted in rapid acute mortality, comparable to that seen in *Myd88*^-/-^ mice (Fig 3A). Analysis of the parasite burden revealed that while TLR11 was the primary mediator of parasite control at the site of infection, Casp1/11 plays an important role in cooperation with TLR11 in providing systemic Myd88-dependent immunity towards this intracellular pathogen (Fig 3B).

The rapid susceptibility of *TLR11*x*Casp1/11*^-/-^ mice suggests that Casp1/11 is required for strong induction of IFN-γ in the absence of TLR11 during parasite infection. This was evident from the analysis of *IFN-γ* expression in peritoneal cavity and spleen of the infected *TLR11*x*Casp1/11*^-/-^ mice (Fig 3C). While both TLR11 and Casp1/11 cooperate in host resistance to *T*. *gondii*, these signaling pathways play distinct roles in programming host defense. We observed that TLR11, but not Casp1/11 played a role in the regulation of systemic IL-12 responses. This was evident from the analysis of IL-12/23p40 detected in the sera of infected *TLR11*^-/-^ and *TLR11*x*Casp1/11*^-/-^ mice (Fig 3D). Consistent with a role for Casp1/11 in the regulation of the circulating IFN-γ, we also observed a significant reduction in serum IFN-γ levels detected in *TLR11*x*Casp1/11*^-/-^ mice compared to TLR11-deficienct animals (Fig 3D). These data strongly suggest that in the absence of TLR11, the Casp1/11-dependent inflammasome pathway significantly contributes to controlling *T*. *gondii* and systemic IFN-γ production during infection.

We then examined if the Casp1/11-dependent cytokines, IL-18 and IL-1β, were playing a compensatory role in the absence of TLR11-mediated IL-12 during *T*. *gondii* infection. We observed on day 5 of parasite infection circulating IL-18 was significantly elevated in *TLR11*^-/-^ mice compared to both WT and *TLR11*x*Casp1/11*^-/-^ mice (S3A Fig). Moreover, *TLR11*^-/-^ mice sustained these elevated levels of IL-18 compared to *TLR11*x*Casp1/11*^-/-^ mice by day 8 post-infection (S3A Fig). Contrariwise, we were unable to detect any circulating IL-1β on days 5 and 8 of *T*. *gondii* infection in WT, *TLR11*^-/-^, *TLR11*x*Casp1/11*^-/-^ mice (S3B Fig). Overall, our results revealed a pivotal role for Casp1/11 in controlling *T*. *gondii* and systemic IFN-γ production in the absence of TLR11.

### TLR11 and the inflammasome are essential for a robust parasite-mediated CD4+ T_H_1 derived IFN-γ response

Among the several classes of innate immune receptors, TLRs are known to play a central role in the regulation of T_H_1 effector choices. At the same time, both IL-1 and IL-18 are known to be required for the sustained IFN-γ production by CD4+ T cells and the combined deficiency in TLR and IL-1R signaling contributes to a compromised T_H_1 immunity seen in Myd88-deficient mice infected with the intracellular pathogens [10].

**Fig 4:**
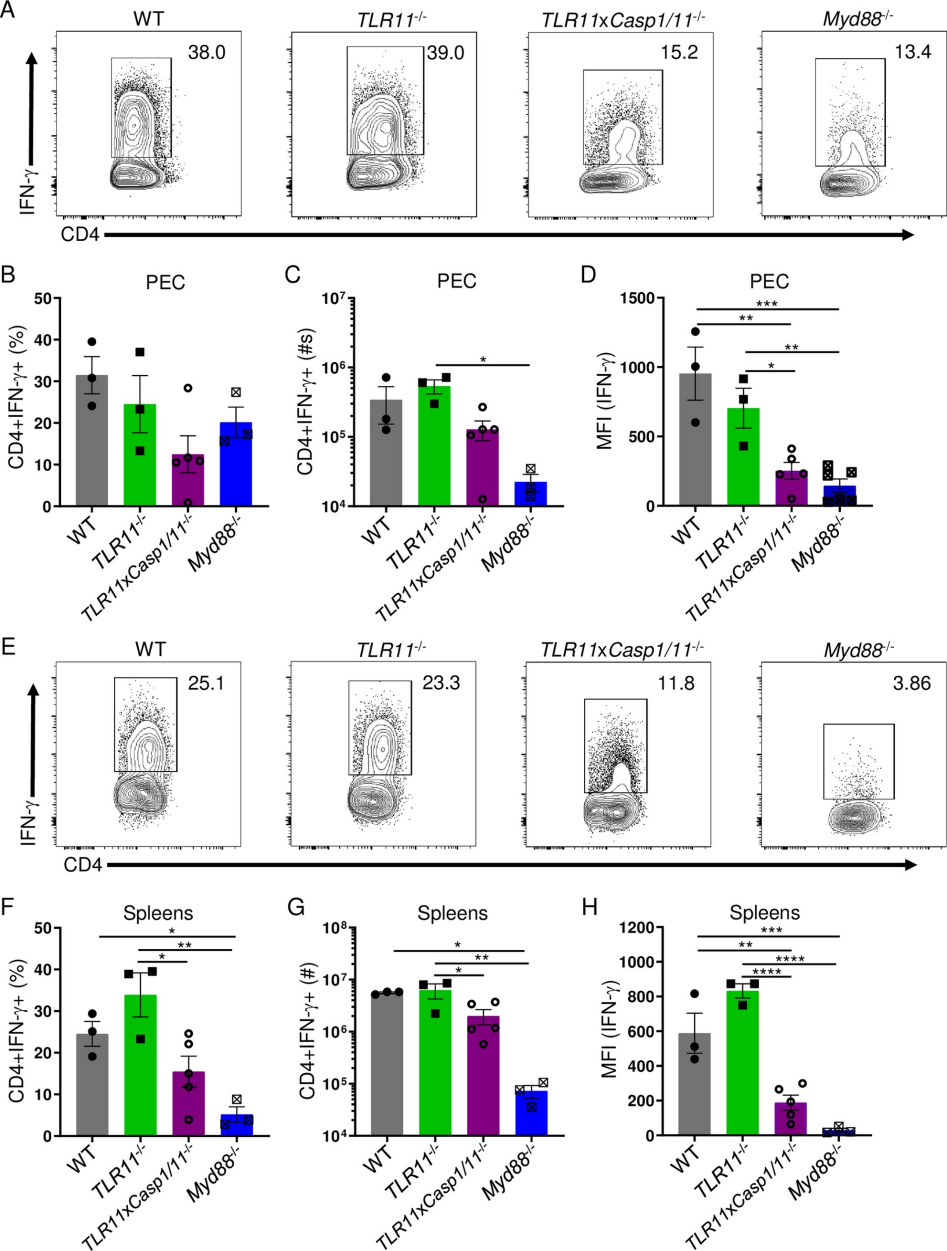
*In the absence of TLR11*, *inflammasome activation is required for robust T*_*H*_*1 responses during* T. gondii *infection*. (**A, E**) WT, *TLR11*^-/-^, *TLR11*x*Casp1/11*^-/-^, and *Myd88*^-/-^ mice were infected i.p. with 20 cysts of *T*. *gondii*. PECs (A) and spleens (E) were harvested and IFN-γ production by CD4+ T cells was analyzed by flow cytometry. (**B, C, F, G**) Average frequencies (B, F) and absolute quantification (C, G) of CD4+IFN-γ+ cells in the PECs and spleens were analyzed on day 8 following infection. (**D, H**) MFI of CD4+ T cell IFN-γ was analyzed on day 8 post-infection. Results are representative of three-independent experiments involving at least 3 mice per group. Statistical analyses were done using one-way ANOVA with a Tukey’s multiple comparison test, *P<0.05, **P<0.01, ***P<0.001, ****P<0.0001. Error bars, standard error mean.

We next investigated the relative contribution of TLR11 and Casp1/11 in the regulation of T_H_1 immunity to *T*. *gondii*. Infection of *TLR11*^-/-^ mice unexpectedly revealed TLR11-deficiency played no obvious role in the initiation of a CD4+ T_H_1 response towards *T*. *gondii*. This was in sharp contrast to TLR11xCasp1/11-deficient mice that demonstrated a profound reduction in both frequency and absolute numbers of CD4+IFN-γ+ T cells at the site of infection (Fig 4A–4C). Furthermore, Casp1/11-deficiency in the absence of TLR11 resulted in a significant reduction of IFN-γ production among peritoneal CD4+ T cells as measured by intracellular staining for this cytokine (Fig 4D). Both the frequencies of CD4+IFN-γ+ and the amounts of IFN-γ produced by CD4+ T cells were similar in *TLR11*x*Casp1/11*^-/-^ and *Myd88*^-/-^ mice (Fig 4B and 4D), strongly suggesting that both of these signaling pathways cooperate in a Myd88-dependent manner that is essential for T_H_1 effector function during *T*. *gondii* infection.

Analogous to our results from the peritoneum, our data showed both frequency and total cell number of splenic CD4+IFN-γ+ T cells, and the amounts of IFN-γ produced by CD4+ T cells were not compromised by a single TLR11- or Casp1/11-deficiency (Figs 2E–2H and 4E–4H). Instead, deficiency in both TLR11 and Casp1/11 resulted in the reduced frequencies and total T_H_1 cells, and the amounts of IFN-γ produced by CD4+ T cells (Fig 4E–4H, S4 Fig). Additionally, in *TLR11*x*Casp1/11*^-/-^ mice we observed a reduction in the absolute numbers of IFN-γ producing peritoneal CD8+ T cells and NK cells, similar to *Myd88*^-/-^ animals (S5A Fig). However, a reduction in the absolute numbers of IFN-γ producing splenic CD8+ T cells and NK cells was only observed in *Myd88*^-/-^ mice (S5B Fig).

Overall, the analysis of TLR11- and Casp1/11-deficient mice allowed us to reveal that inflammasome activation plays a major role in the regulation of T_H_1 immunity when TLR11-dependent recognition is compromised. Our results also demonstrated that *T*. *gondii* activation by the inflammasome Casp1/11-dependent pathway is sufficient for Myd88-dependent activation of T_H_1 immunity. This provided an explanation for largely unimpaired activation of CD4+ T cells observed in TLR11-deficient mice infected with the parasite.

### IL-18 is sufficient and necessary for robust CD4+ T_H_1-derived IFN-γ during *T*. *gondii* infection in the absence of TLR11

**Fig 5:**
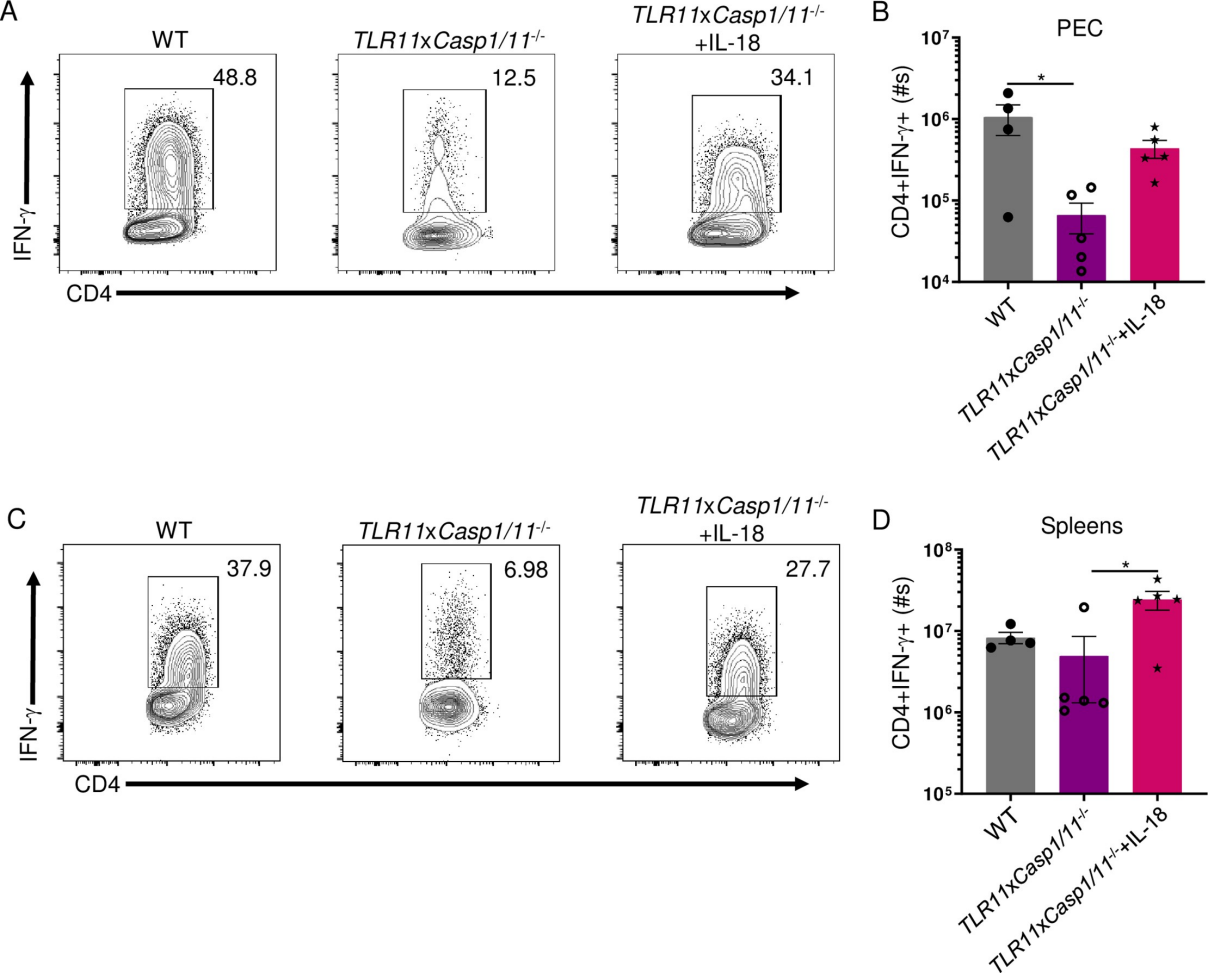
IL-18 is sufficient to augment T_H_1-derived IFN-γ responses in the absence of TLR11. (**A, C**) WT and *TLR11*x*Casp1/11*^-/-^ mice were infected with 20 cysts of *T*. *gondii*. *TLR11*x*Casp1/11*^-/-^ mice were administered 200 ngs of IL-18 i.p. on days 0, 1, 2, and 3. PECs (A) and spleens (C) were harvested and IFN-γ production by CD4+ T cells was analyzed by flow cytometry. (**B**, **D**) Absolute quantification of CD4+IFN-γ+ cells in the PECs (B) and spleens (D) were analyzed on day 8 following infection. Results are representative of three-independent experiments involving at least 3 mice per group. Statistical analyses were done using one-way ANOVA with a Tukey’s multiple comparison test, *P<0.05. Error bars, standard error mean.

Inflammasome recognition of intracellular pathogens is essential for the release of mature forms of IL-1β and IL-18. The cytokine IL-18 is critical for the production and secretion of IFN-γ by immune cells [36, 38, 40]. Therefore, we hypothesized in the absence of TLR11, IL-18 is required for robust CD4+ T_H_1-derived IFN-γ responses during parasite infection. To determine if the compromised CD4+ T cell-derived IFN-γ response of *TLR11*x*Casp1/11*^-/-^ mice is a result of IL-18 deficiency, we neutralized either IL-1β or IL-18 in *TLR11*^-/-^ mice during *T*. *gondii* infection. Our results revealed IL-1β does not significantly contribute to the amounts of IFN-γ produced by T_H_1s in TLR11-deficient mice (S6 Fig). Simultaneously, antibody-mediated blocking of IL-18 in *TLR11*^-/-^ mice resulted in a dramatic reduction in the amounts of IFN-γ produced by both peritoneal and splenic CD4+ T cells, similar to *TLR11*x*Casp1/11*^-/-^ mice (S6 Fig). To confirm IL-18 is critical to augment CD4+ T cell-derived IFN-γ production in the absence of TLR11, we administered IL-18 to *TLR11*x*Casp1/11*^-/-^ mice during *T*. *gondii* infection and examined their CD4+ T_H_1 responses. Our results revealed administering IL-18 to *TLR11*x*Casp1/11*^-/-^ mice during parasite infection dramatically augmented the frequency and absolute numbers of CD4+IFN-γ+ T cells both locally in the peritoneum and in the spleen, compared to non-treated controls (Fig 5, S7 Fig). Our data also demonstrate that *TLR11*x*Casp1/11*^-/-^ mice given IL-18 during infection augments the absolute numbers of peritoneal and splenic CD8+IFN-γ+ T cells, IFN-γ+ NK cells, and IFN-γ+ neutrophils (S8A and S8B Fig). Finally, our results demonstrate that IL-18 does contribute in reducing parasite burden both at the site of infection and in peripheral tissue (S8C Fig), but failed to rescue the survival of *TLR11*x*Casp1/11*^-/-^ mice infected with the parasite (S8D Fig). Our data revealed that in the absence of TLR11, inflammasome activation and IL-18 is sufficient and necessary in augmenting peritoneal and splenic IFN-γ production by CD4+ and CD8+ T cells along with NK cells during *T*. *gondii* infection and limiting pathogen burden. Nevertheless, other Casp1/11-dependent cytokines are required for host survival during *T*. *gondii* infection.

## Discussion

Activation of inflammasomes by pathogen stimuli recognition results in the oligomerization and recruitment of the adaptor molecule ASC, caspase-1 activation, release of the active forms of IL-1β and IL-18, and induction of the cell-mediated death pathway, pyroptosis. Accordingly, pathogen ligand recognition by inflammasomes is considered an essential host innate immune pathway to identify invading microbes. Recent reports have demonstrated that *T*. *gondii* is recognized by the NLRP1 and NLRP3 inflammasomes resulting in IL-1β and IL-18 production both *in vitro* and *in vivo* [26, 32]. It has also been established that TLR recognition of the parasite by DCs is required for IL-12 production [16, 18, 20]. The *T*. *gondii*-driven IL-12 production is essential for generating a robust CD4+ T_H_1-derived IFN-γ response, resulting in the induction of IFN-γ-mediated genes required for parasite clearance [11, 12, 41–43]. Additionally, previous reports have shown that the inflammasome-dependent cytokine IL-18 can work in synergy with IL-12 during *T*. *gondii* infection, augmenting IFN-γ responses and contributing to parasite restriction [32, 37, 38]. Hence, we hypothesized that a deficiency in NLRP3, ASC, or Casp1/11 would significantly abrogate CD4+ T cell-derived IFN-γ responses, resulting in rapid host mortality. Unexpectedly, our results did not support the original hypothesis, instead revealing that inflammasome-deficient mice do not have increased mortality rates during parasite infection and only have a modest reduction of IL-12 and IFN-γ. Additionally, we observed no change in the frequency or absolute cell numbers of peritoneal or splenic CD4+ T_H_1 cells. Thus, our data demonstrates that the inflammasome plays a limited role in parasite restriction and is dispensable for murine host resistance against *T*. *gondii* when TLR11-mediated immunity remains intact.

Two previous studies have reported contradicting results as to the role of the inflammasome in host immunity towards *T*. *gondii*. The data reported by Hitziger and colleagues are consistent with our own results, indicating inflammasome deficiency alone does not play a major role in parasite restriction [44]. However, Gorfu and colleagues’ data indicate that the absence of either the NLRP1 or NLRP3 inflammasome results in increased parasite burden and host mortality [32]. While additional work is required to resolve these inconsistencies, a largely dispensable role for caspase-1 and -11 in the survival of *T*. *gondii* infected mice strongly suggest a limited role for inflammasomes alone in host resistance to the parasite when TLR11-sensing remains intact.

Innate recognition of *T*. *gondii* by TLRs is critical for robust IL-12 production and host defense. Consequently, it has been shown that TLR11 and TLR12, the only known innate receptors to uniquely recognize *T*. *gondii* profilin and who significantly contribute to IL-12 production, are not absolutely required for host resistance or for T_H_1 effector function [16, 18, 20]. Nevertheless, it is important to note that we observed a higher susceptibility of *TLR11*^-/-^ mice than previously reported [16], as on average half of the infected mice succumb to *T*. *gondii* during an acute stage of the infection. Considering that the same ME49 parasites were used in both studies, the difference in susceptibility to *T*. *gondii* may in part be caused by distinct microbiota that regulate immunity to the parasite and other pathogens [45, 46].

Analysis of UNC93B1-deficient mice, which lack all endosomal TLR signaling, including TLR11 and TLR12, resulted in diminished IL-12 responses and a delayed *T*. *gondii*-mediated IFN-γ response. Yet, this delayed TLR-independent parasite-driven IFN-γ response is insufficient for host protection [18, 20]. Host immunity in these TLR or UNC93B1 knockouts can be rescued by administering exogenous IL-12 [18, 20]. Paradoxically, *Myd88*-deficient mice, are not only highly susceptible to parasite infection, but cannot be rescued with exogenous administration of IL-12, strongly suggesting that signaling from both IL-12 and the inflammasome-mediated cytokines IL-1β and IL-18 are required for host resistance against *T*. *gondii* [10]. Furthermore, it has been reported that both IL-1β and IL-18 play a role in augmenting IFN-γ production during *T*. *gondii* infection [37, 47]. Hence, we hypothesized that in the absence of TLR recognition, inflammasome activation is required for host resistance against *T*. *gondii*. The results from *TLR11*x*Casp1/11*^-/-^ mice demonstrated that in the absence of both robust IL-12 and IL-18 production, CD4+ T cell-derived IFN-γ responses were compromised and insufficient for host protection resulting in uncontrolled parasite replication. Therefore, since *TLR11*x*Casp1/11*^-/-^ mice have intact IL-18R signaling, we examined if exogenous IL-18 administered to *TLR11*x*Casp1/11*^-/-^ mice during parasite infection would be sufficient to restore CD4+ T_H_1-derived IFN-γ responses. Our results demonstrated that in the absence of TLR11, IL-18 is sufficient and necessary for robust CD4+ T cell-derived IFN-γ responses. Hence, our data establishes two significant attributes of inflammasome activation during parasite infection: 1) in the absence of TLR11 recognition, the inflammasome and IL-18 is critical for robust T_H_1 effector function; and 2) they provide pivotal evidence as to why IL-12 alone is insufficient to rescue *Myd88*^-/-^ mice during *T*. *gondii* infection.

Our findings illustrate the mechanism by which triggering inflammasome activation in the absence of TLR11 enables host resistance against *T*. *gondii* infection. Dissimilar to murine innate recognition of *T*. *gondii*, humans lack a functional TLR11, and TLR12 is absent from the human genome [48]; however, parasite infection in immunocompetent individuals is generally asymptomatic, indicating TLR11- and TLR12-independent innate recognition of *T*. *gondii* is sufficient for human immunity against the parasite. Both CCL2-dependent recruitment of human monocytes and NLRP1, NLRP3, ASC, and caspase-1-dependent release of IL-1β and IL-18 by these cells suggest that *T*. *gondii*-triggered activation of the inflammasome is required for resistance against the parasite when TLR11-dependent immunity is absent [24, 28, 49].

Unlike for TLR11 and TLR12, the precise *T*. *gondii* ligand that is recognized by inflammasomes remains unknown. However, reports have demonstrated that heat killed *T*. *gondii* or mycalolide B-treated parasites are unable to trigger IL-1β release, indicating that active parasite invasion is required for inflammasome-mediated cytokine release [24, 32]. Intriguingly, while it has been demonstrated that *T*. *gondii* induces both pyroptosis and IL-1β secretion in rat macrophages, mouse macrophages do not undergo pyroptosis during parasite invasion *in vitro* [27, 50]. Additionally, reports have indicated that predominantly type II strains of the parasite, such as ME49, which was used in this current study, promote substantial cytokine release compared to the majority of other identified *T*. *gondii* strains [24, 32]. It has also been shown that the dense granule protein, GRA15, from type II strains of the parasite plays an important role for IL-1β release in both human monocytes and mouse bone marrow-derived macrophages [24, 32]. It has yet to be determined if GRA15 is directly recognized by inflammasome sensors resulting in caspase-1 activation and IL-1β release; or if GRA15, a known inducer of NF-κB activation, leads to the induction of immature IL-1β and a second *T*. *gondii*-dependent stimulus triggers caspase-1-dependent cytokine release [51]. Furthermore, recent reports show that *T*. *gondii*-mediated potassium efflux can trigger the rapid release of IL-1β in infected monocytes [28]. While the precise *T*. *gondii* stimuli that triggers inflammasome activation remains unknown, our results established that inflammasome activation and IL-18 are required for host protection and robust CD4+ T cell-derived IFN-γ responses in the absence of TLR11.

## Materials and methods

### Animals

C57BL/6, *Nlrp3*^-/-^, *and Casp1/11*^-/-^ mice were obtained from Jackson Laboratory (Bar Harbor, ME). *Asc*^-/-^, *TLR11*^-/-^ and *Myd88*^-/-^ mice have been previously described [16, 52, 53]. *TLR11*^-/-^ mice were crossed with *Casp1/11*^-/-^ mice to generate *TLR11*x*Casp1/11*^-/-^. All control and experimental mice were age- and sex-matched within all individual experiments. This study included both male and female mice, and the data derived from male and female mice identified no sex-specific differences in the performed experiments. All mice were maintained at in the pathogen-free American Association of Laboratory Animal Care-accredited animal facility at the University of Rochester Medical Center, Rochester, NY. All animal experimentation was conducted in accordance with the guidelines of the University of Rochester’s University Committee on Animal Resources (UCAR), the Institutional Animal Care and Use Committee (IACUC).

### *Toxoplasma gondii* infection and RT-PCR

All mice were i.p. infected with an average of 20 *T*. *gondii* cysts of the ME49 strain. At day 8 post-infection, the animals were necropsied. In some experiments, mice were injected i.p. with 200 ng of IL-18 (R&D) on days 0, 1, 2, and 3, or mice were injected with 200 μgs of anti-IL-1β or anti-IL-18 (BioXCell) on days 0, 2, 4, and 6. Total RNA was isolated from the peritoneal exudate cells and the spleens of naïve or *T*. *gondii* infected mice using Trizol. cDNA was prepared using iScript cDNA Synthesis Kit (Bio-Ras, Hercules, CA). Optimized primers targeting each gene were designed using Primer3 Software [54]. These primers included the following: *IFN-γ* (5’-ACTGGCAAAAGGATGGTGAC-3’, 3’-TGAGCTCATTGAATGCTTGG-5’), *Irgm3* (5’-CTGGAGGCAGCTGTCAGCTCCGAG-3’, 3’-GTCCTTTAGAGCTTTCCTCAGGGAGGTCTTG-5’), *Cxcl10* (5’-GACGGTCCGCTGCAACTG-3’, 3’-GCTTCCCTATGGCCCTCATT-5’), and *HPRT* (5’-gcccttgactataatgagtacttcagg-3’, 5’-ttcaacttgcgctcatcttagg-3’) [55]. cDNA was amplified using SSOFast Eva Green Supermix (BioRad). A MyiQ Real-Time PCR Detection System (BioRad) was used to obtain Ct values. The relative expression of samples was determined after normalization to *HPRT* using ddCt method [56].

To determine *T*. *gondii* pathogen loads, total genomic DNA from animal tissue was isolated by using the DNeasy Blood and Tissue Kit (Qiagen) according to manufacturer’s instructions. PCR were performed by using SSOFast Eva Green Supermix (BioRad). Samples were measured by qPCR using a MyiQ Real-Time PCR Detection System (BioRad), and data from genomic DNA was compared with a defined copy number standard of the T. *gondii* gene *B1*.

### ELISA Analysis

The IFN-γ (ThermoFisher), IL-12/23p40 (ThermoFisher), IL-1β (ThermoFisher), and IL-18 (R&D) concentration in the sera or cell supernatant was analyzed by standard sandwich ELISA kit according to manufacturer’s instructions.

### Measurements of CD4+ T cell responses

To assay the responses of mice infected with *T*. *gondii* the PECs and spleens were harvested from C57BL/6, *Nlrp3*^-/-^, *Asc*^-/-^, *Casp1/11*^-/-^, *TLR11*^-/-^, *TLR11*x*Casp1/11*^-/-^, and *Myd88*^-/-^ mice and on day 8 post-infection. Single-cell suspension of PECs and spleens were restimulated with PMA (20ng/mL) and ionomycin (1μg/mL) (Sigma-Aldrich) for 4 hours in the presence of GolgiPlug (Brefeldin A, BD Biosciences). Alternatively, bone marrow-derived DCs (BMDCs) were generated in the presence of GM-CSF (Invitrogen) and plated at 2.5x10^6^ cells/well in a 96-well plate and pulsed with frozen ME49 tachyzoite antigen for 18 hours and then 2.5x10^6^ splenic T cells were added for 6 hours in the presence of GolgiPlug. After isolation or *in vitro* restimulation, the cells were washed once in phosphate-buffered saline + 1% fetal bovine serum and stained with fluorochrome-conjugated antibodies. Cell fluorescence was measured using an LSRII flow cytometer, and data were analyzed using FlowJo Software (Tree Star).

### Statistical analysis

All data were analyzed with Prism (Version 7; GraphPad) These data were considered statistically significant when *P*-values were <0.05.

## Supporting information

S1 Fig Inflammasome activation is dispensable to control *T. gondii* cysts. WT, *Nlrp3*^-/-^, *Asc*^-/-^, *Casp1/11*^-/-^, *TLR11*^-/-^ mice were i.p. infected with 20 cysts of *T*. *gondii*. Cysts count in the brain determined at 30 days post-infection. (**B, C**) Analysis by ELISA of serum IL-12/23p40 and IFN-γ in WT, *Nlrp3*^-/-^, *Asc*^-/-^, and *Casp1/11*^-/-^ mice infected with *T*. *gondii* was performed on day 8 post-infection. (**D-F**) qRT-PCR analysis of relative *IFN-γ* (D), *Irgm3*, and *Cxcl10* expression measured in the PECs (E) and spleens (F) of mice infected with 20 cysts of ME49 on day 8 post-infection. Results are representative of three-independent experiments involving at least 3 mice per group. Statistical analyses were done using one-way ANOVA with a Tukey’s multiple comparison test, *P<0.05, **P<0.01, ***P<0.001, ****P<0.0001. Error bars, standard error mean.
(TIF)

S2 Fig *T. gondii*-mediated IFN-γ responses from CD8+ T cells, NK cells, and neutrophils are inflammasome-independent. WT, *Nlrp3*^-/-^, *Asc*^-/-^, and *Casp1/11*^-/-^ mice were i.p. infected with 20 cysts of *T*. *gondii*. Absolute quantification of CD8+IFN-γ+, NK1.1+IFN-**γ**+, and Ly6G+IFN-**γ**+ cells in the PECs (**A**) and spleens (**B**) were analyzed on day 8 following infection. Results are representative of three-independent experiments involving at least 3 mice per group. Statistical analyses were done using one-way ANOVA with a Tukey’s multiple comparison test, *P<0.05. Error bars, standard error mean.
(TIF)

S3 Fig The absence of TLR11 and Casp1/11 results in reduced IL-18 production during *T. gondii* infection. Analysis by ELISA of serum IL-18 and IL-1β in WT, *TLR11*^-/-^, and *TLR11*x*Casp1/11*^-/-^ mice infected with *T*. *gondii* was performed on days 5 and 8 of infection. Results are representative of two-independent experiments. Results are representative of three-independent experiments involving at least 3 mice per group. Statistical analyses were done using one-way ANOVA with a Tukey’s multiple comparison test, **P<0.01. Error bars, standard error mean.
(TIF)

S4 Fig TLR11xCasp1/11-deficiency reduces CD4+ T_H_1-derived IFN-γ production to restimulation with *T. gondii* antigen. WT, *TLR11*^-/-^, and *TLR11*x*Casp1/11*^-/-^ mice were i.p. infected with 20 cysts of ME49 *T*. *gondii*. Splenic T cells were harvested, added to BMDCs pulsed overnight with frozen ME49 antigen, and IFN-γ production by CD4+ T cells was analyzed by flow cytometry. Absolute quantification of splenic CD4+IFN-γ+ cells were analyzed on day 8 following infection. Results are representative of three-independent experiments involving at least 3 mice per group. Statistical analyses were done using one-way ANOVA with a Tukey’s multiple comparison test, *P<0.05. Error bars, standard error mean.
(TIF)

S5 Fig IFN-γ responses from peritoneal CD8+ T cells and NK cells, are reduced in *TLR11xCasp1/11^-/-^* mice. WT, *TLR11*^-/-^, *TLR11*x*Casp1/11*^-/-^, and *Myd88*^-/-^ mice were infected i.p. with 20 cysts of *T*. *gondii*. Absolute quantification of CD8+IFN-γ+, NK1.1+IFN-γ+, and Ly6G+IFN-γ+ cells in the PECs (**A**) and spleens (**B**) were analyzed on day 8 following infection. Results are representative of three-independent experiments involving at least 3 mice per group. Statistical analyses were done using one-way ANOVA with a Tukey’s multiple comparison test, *P<0.05. Error bars, standard error mean.
(TIF)

S6 Fig During acute *T. gondii* infection IL-1β is dispensable, but IL-18 plays an important role in contributing to CD4+ T cell-derived IFN-γ in the absence of TLR11. WT, *TLR11*^-/-^, and *TLR11*x*Casp1/11*^-/-^ mice were infected i.p. with 20 cysts of *T*. *gondii*. *TLR11*^-/-^ mice were administered 200 μgs of either anti-IL-1β or anti-IL-18 i.p. on days 0, 2, 4, and 6. PECs (**A**) and spleens (**B**) were harvested and IFN-γ production by CD4+ T cells was analyzed by flow cytometry. MFI of CD4+ T cell IFN-γ was analyzed on day 8 post-infection. Results are representative of three-independent experiments involving at least 3 mice per group. Statistical analyses were done using one-way ANOVA with a Tukey’s multiple comparison test. Error bars, standard error mean.
(TIF)

S7 Fig IL-18 augments the production of IFN-γ by CD4+ T cells in TLR11- and Casp1/11-deficient hosts. (**A**, **B**) WT and *TLR11*x*Casp1/11*^-/-^ mice were infected with 20 cysts of *T*. *gondii*. *TLR11*x*Casp1/11*^-/-^ mice were administered 200 ngs of IL-18 i.p. on days 0, 1, 2, and 3. PECs (A) and spleens (B) were harvested and IFN-γ production by CD4+ T cells was analyzed by flow cytometry. Average frequencies of CD4+IFN-γ+ cells in the PECs (A) and spleens (B) were analyzed on day 8 following infection. (A, B) MFI of CD4+ T cell IFN-γ was analyzed on day 8 post-infection. Results are representative of three-independent experiments involving at least 3 mice per group. Statistical analyses were done using one-way ANOVA with a Tukey’s multiple comparison test, *P<0.05, **P<0.01. Error bars, standard error mean.
(TIF)

S8 Fig IL-18 augments IFN-γ responses from peritoneal CD8+ T cells, NK cells, and neutrophils in *TLR11xCasp1/11^-/-^* mice. WT, *TLR11*x*Casp1/11*^-/-^, mice were infected i.p. with 20 cysts of *T*. *gondii*. *TLR11*x*Casp1/11*^-/-^ mice were administered 200 ngs of IL-18 i.p. on days 0, 1, 2, and 3. Absolute quantification of CD8+IFN-γ+, NK1.1+IFN-γ+, and Ly6G+IFN-γ+ cells in the PECs (**A**) and spleens (**B**) were analyzed on day 8 following infection. (**C**) Analysis of *T*. *gondii* parasite loads by qPCR from PECs and spleens on day 8 of infection. Results are representative of three-independent experiments involving at least 3 mice per group. (**D**) Statistical analyses were done using one-way ANOVA with a Tukey’s multiple comparison test, *P<0.05, **P<0.01. Error bars, standard error mean. Survival of WT, *Casp1/11*^-/-^, *TLR11*^-/-^, *TLR11*x*Casp1/11*^-/-^, and *TLR11*x*Casp1/11*^-/-^ mice treated with 200 ngs of IL-18 i.p. on days 0, 1, 2, and 3 post infection. All mice were i.p. infected with 20 cysts of the ME49 strain of *T*. *gondii*. Survival curve is representative of three-independent experiments involving at least 5 mice per group. Statistical analyses of survival curve was done using Log-Rank (Mantel Cox) Test, *P<0.05, **P<0.01, ***P<0.001, ****P<0.0001.
(TIF)
