# Supplementary figures and images for: TLR11-independent inflammasome activation is critical for CD4+ T cell-derived IFN-γ production and host resistance to Toxoplasma gondii
Source: PLoS Pathog. 2019 Jun 13;15(6):e1007872. doi: 10.1371/journal.ppat.1007872 (PMC6599108; doi:10.1371/journal.ppat.1007872)

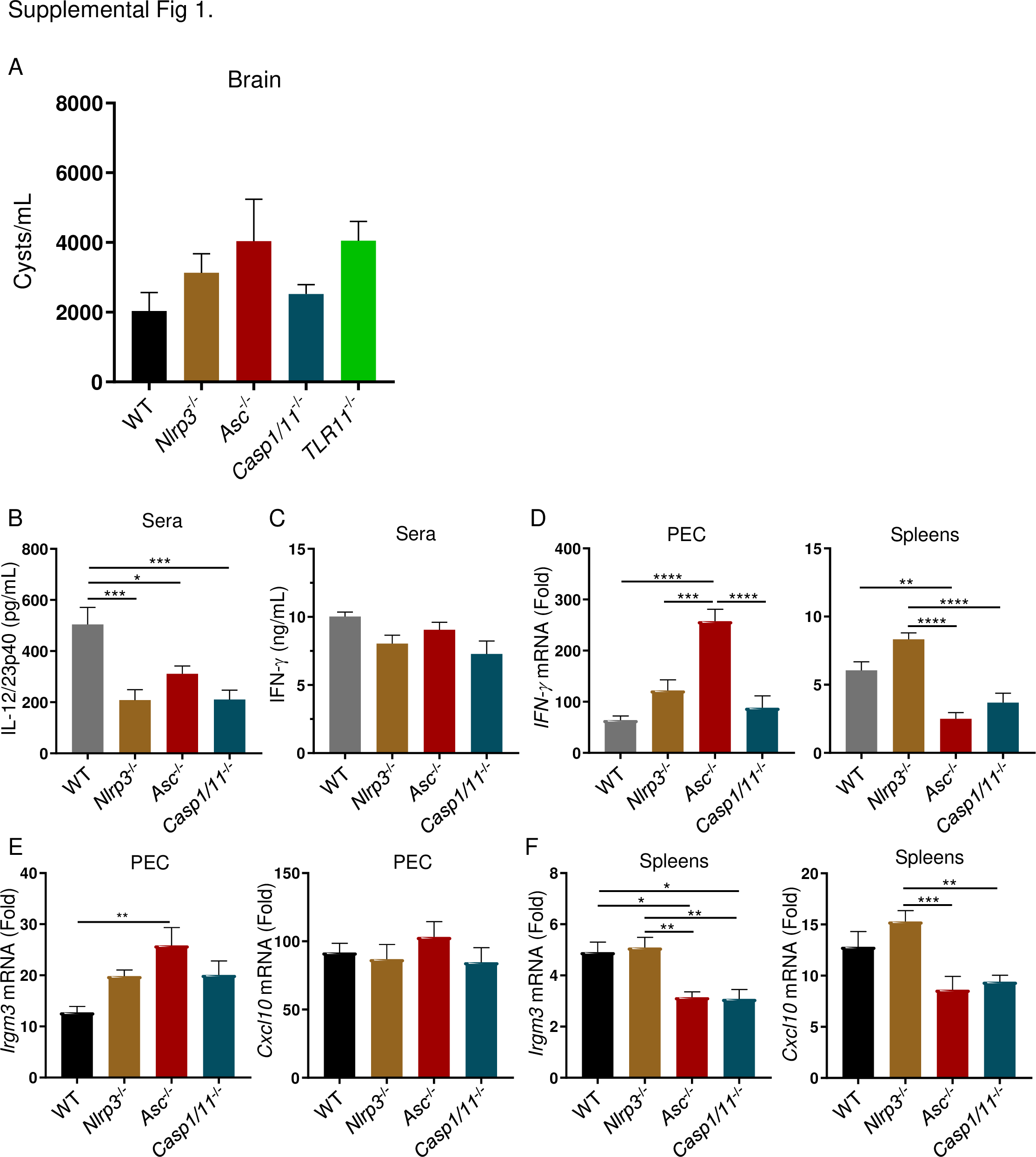

Supplement: S1 Fig — WT, Nlrp3-/-, Asc-/-, Casp1/11-/-, TLR11-/- mice were i.p. infected with 20 cysts of T. gondii. Cysts count in the brain determined at 30 days post-infection. (B, C) Analysis by ELISA of serum IL-12/23p40 and IFN-γ in WT, Nlrp3-/-, Asc-/-, and Casp1/11-/- mice infected with T. gondii was performed on day 8 post-infection. (D-F) qRT-PCR analysis of relative IFN-γ (D), Irgm3, and Cxcl10 expression measured in the PECs (E) and spleens (F) of mice infected with 20 cysts of ME49 on day 8 post-infection. Results are representative of three-independent experiments involving at least 3 mice per group. Statistical analyses were done using one-way ANOVA with a Tukey’s multiple comparison test, *P<0.05, **P<0.01, ***P<0.001, ****P<0.0001. Error bars, standard error mean. (TIF) [file ppat.1007872.s001.tif]

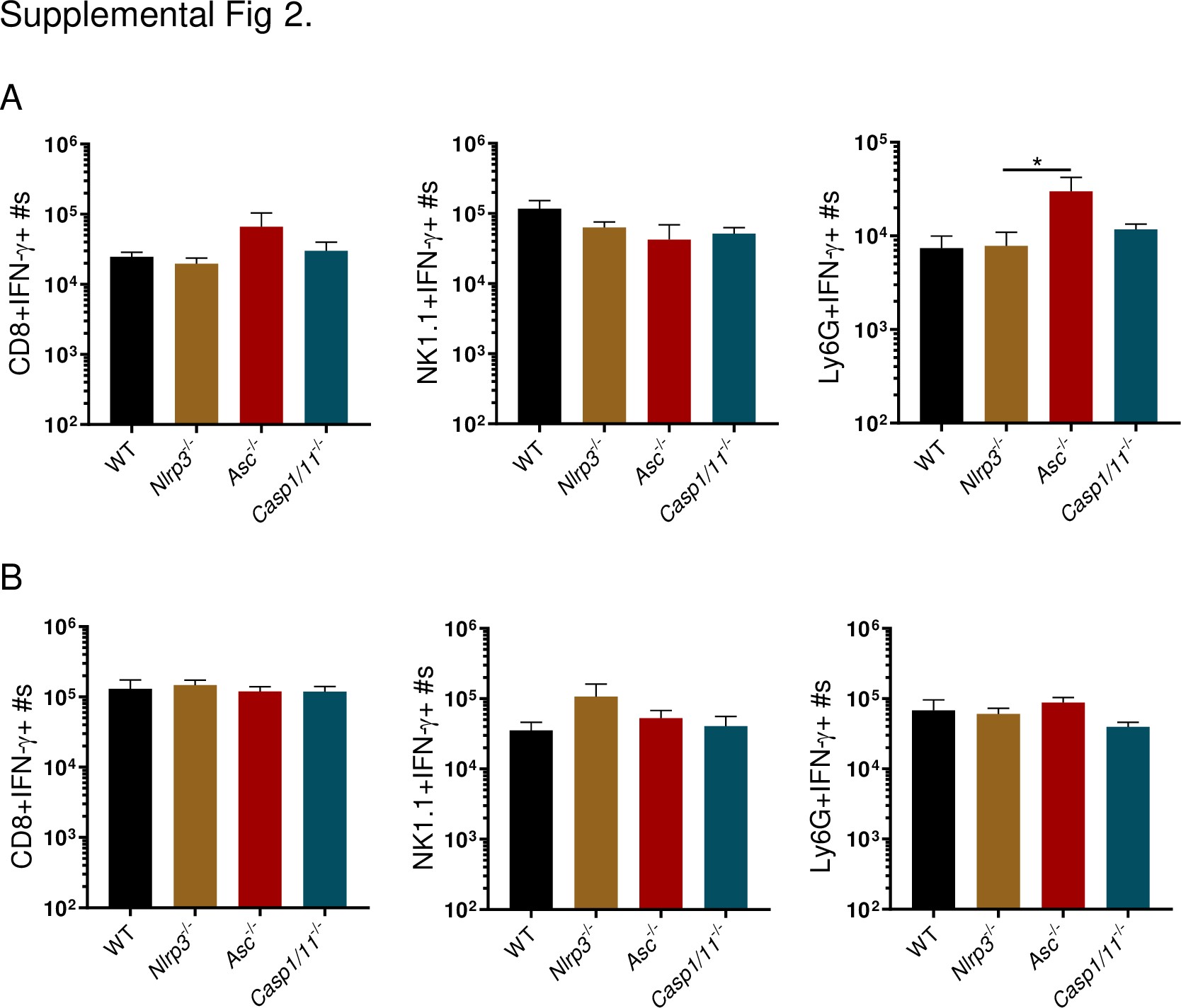

Supplement: S2 Fig — WT, Nlrp3-/-, Asc-/-, and Casp1/11-/- mice were i.p. infected with 20 cysts of T. gondii. Absolute quantification of CD8+IFN-γ+, NK1.1+IFN-γ+, and Ly6G+IFN-γ+ cells in the PECs (A) and spleens (B) were analyzed on day 8 following infection. Results are representative of three-independent experiments involving at least 3 mice per group. Statistical analyses were done using one-way ANOVA with a Tukey’s multiple comparison test, *P<0.05. Error bars, standard error mean. (TIF) [file ppat.1007872.s002.tif]

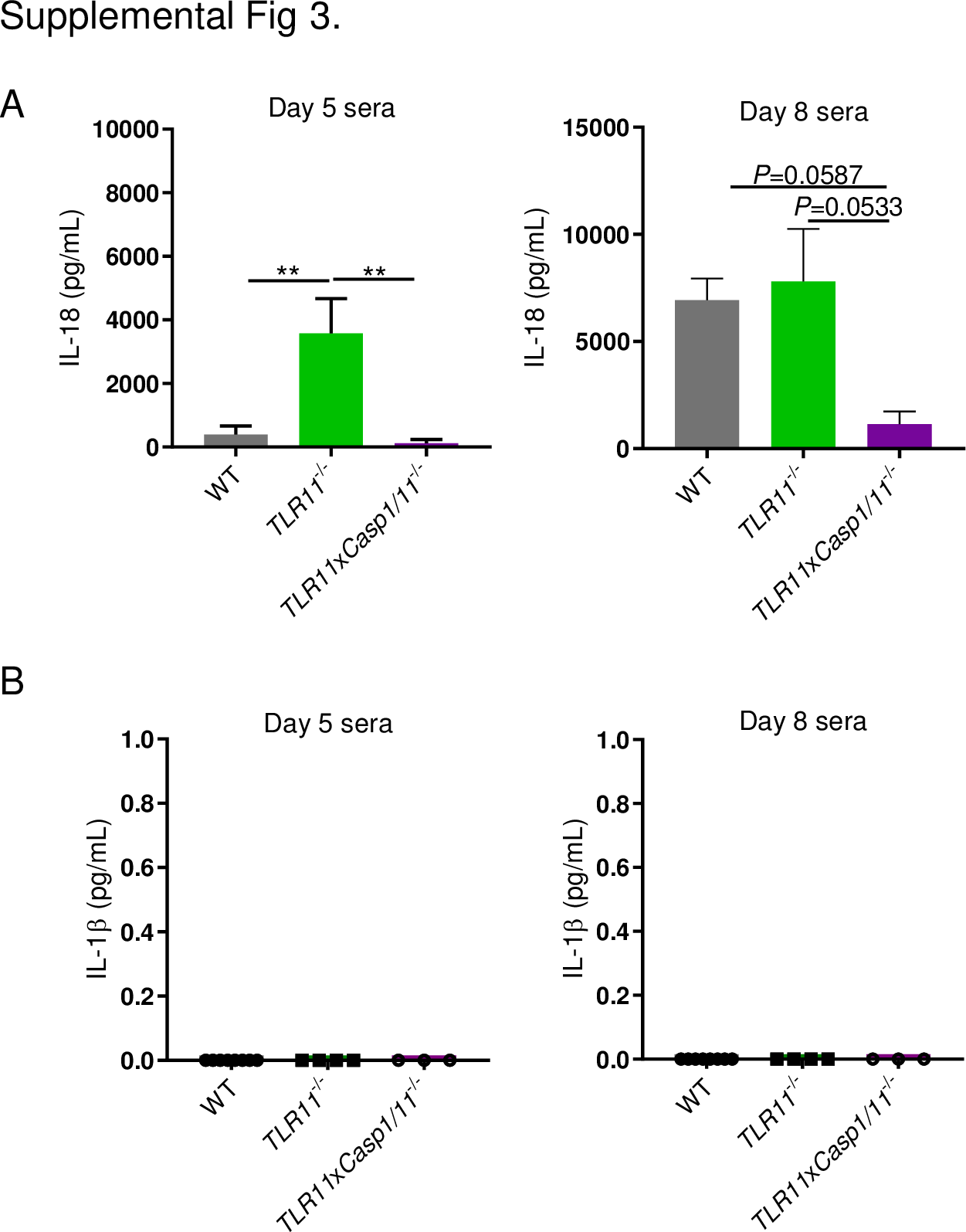

Supplement: S3 Fig — Analysis by ELISA of serum IL-18 and IL-1β in WT, TLR11-/-, and TLR11xCasp1/11-/- mice infected with T. gondii was performed on days 5 and 8 of infection. Results are representative of two-independent experiments. Results are representative of three-independent experiments involving at least 3 mice per group. Statistical analyses were done using one-way ANOVA with a Tukey’s multiple comparison test, **P<0.01. Error bars, standard error mean. (TIF) [file ppat.1007872.s003.tif]

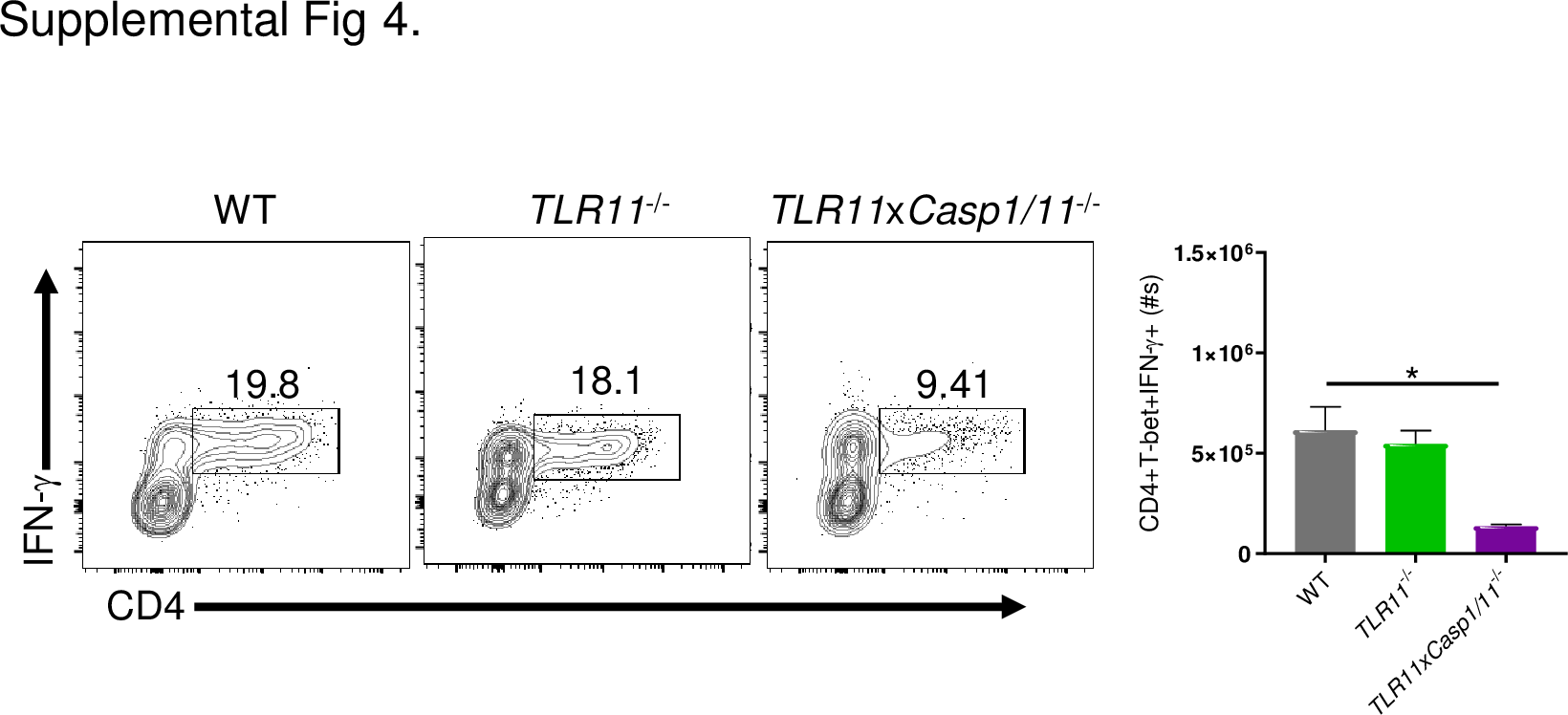

Supplement: S4 Fig — WT, TLR11-/-, and TLR11xCasp1/11-/- mice were i.p. infected with 20 cysts of ME49 T. gondii. Splenic T cells were harvested, added to BMDCs pulsed overnight with frozen ME49 antigen, and IFN-γ production by CD4+ T cells was analyzed by flow cytometry. Absolute quantification of splenic CD4+IFN-γ+ cells were analyzed on day 8 following infection. Results are representative of three-independent experiments involving at least 3 mice per group. Statistical analyses were done using one-way ANOVA with a Tukey’s multiple comparison test, *P<0.05. Error bars, standard error mean. (TIF) [file ppat.1007872.s004.tif]

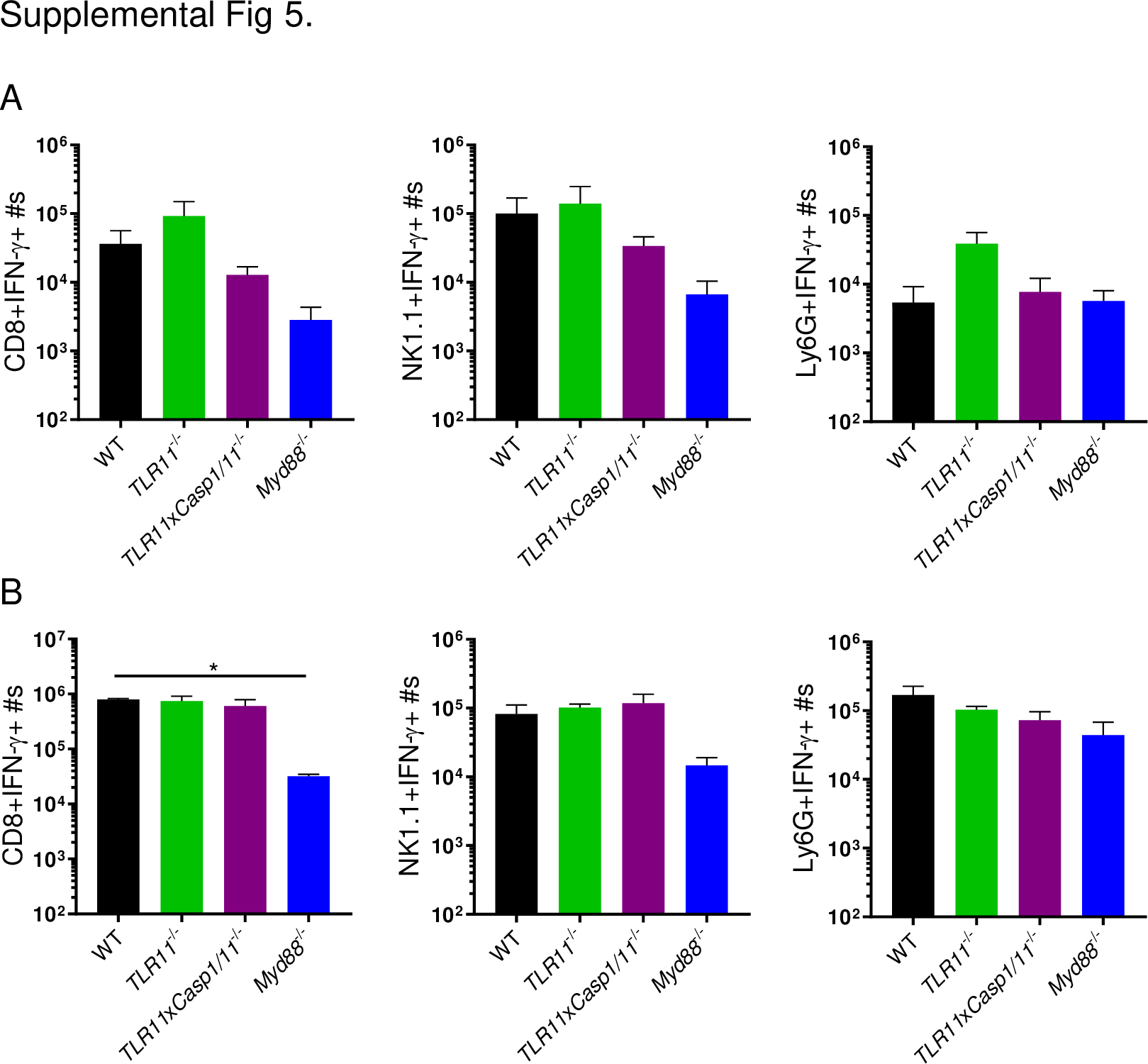

Supplement: S5 Fig — WT, TLR11-/-, TLR11xCasp1/11-/-, and Myd88-/- mice were infected i.p. with 20 cysts of T. gondii. Absolute quantification of CD8+IFN-γ+, NK1.1+IFN-γ+, and Ly6G+IFN-γ+ cells in the PECs (A) and spleens (B) were analyzed on day 8 following infection. Results are representative of three-independent experiments involving at least 3 mice per group. Statistical analyses were done using one-way ANOVA with a Tukey’s multiple comparison test, *P<0.05. Error bars, standard error mean. (TIF) [file ppat.1007872.s005.tif]

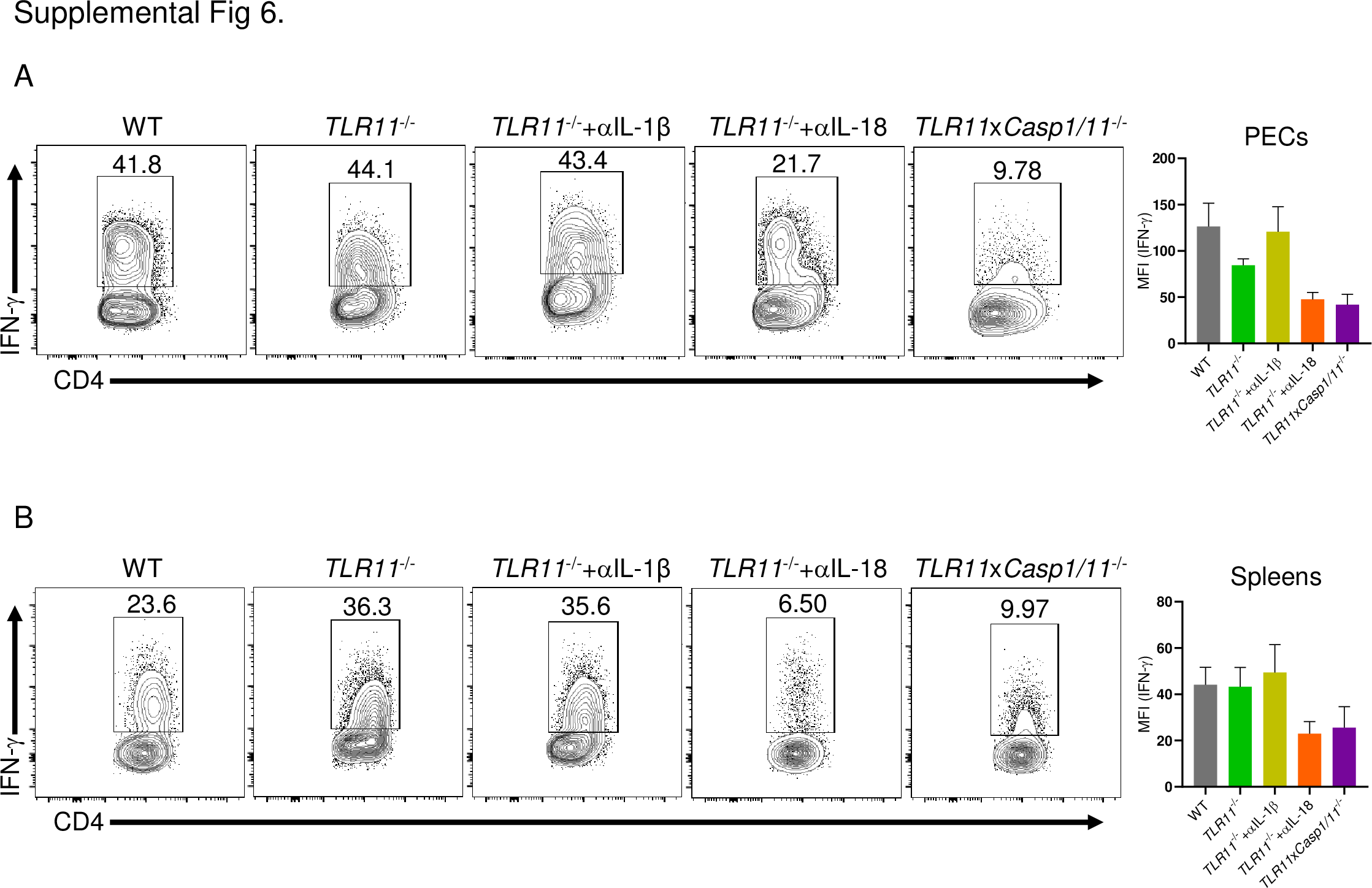

Supplement: S6 Fig — WT, TLR11-/-, and TLR11xCasp1/11-/- mice were infected i.p. with 20 cysts of T. gondii. TLR11-/- mice were administered 200 μgs of either anti-IL-1β or anti-IL-18 i.p. on days 0, 2, 4, and 6. PECs (A) and spleens (B) were harvested and IFN-γ production by CD4+ T cells was analyzed by flow cytometry. MFI of CD4+ T cell IFN-γ was analyzed on day 8 post-infection. Results are representative of three-independent experiments involving at least 3 mice per group. Statistical analyses were done using one-way ANOVA with a Tukey’s multiple comparison test. Error bars, standard error mean. (TIF) [file ppat.1007872.s006.tif]

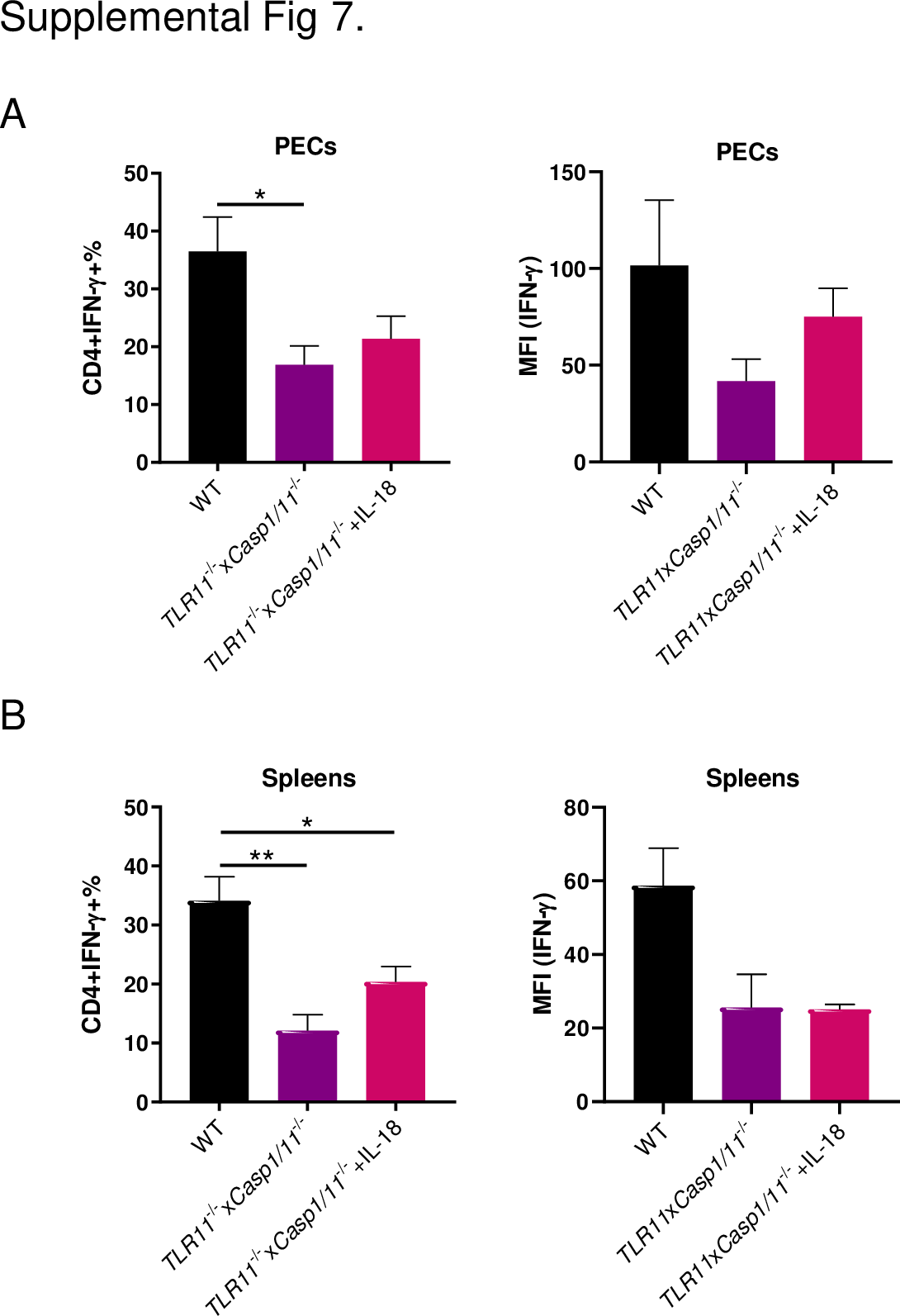

Supplement: S7 Fig — (A, B) WT and TLR11xCasp1/11-/- mice were infected with 20 cysts of T. gondii. TLR11xCasp1/11-/- mice were administered 200 ngs of IL-18 i.p. on days 0, 1, 2, and 3. PECs (A) and spleens (B) were harvested and IFN-γ production by CD4+ T cells was analyzed by flow cytometry. Average frequencies of CD4+IFN-γ+ cells in the PECs (A) and spleens (B) were analyzed on day 8 following infection. (A, B) MFI of CD4+ T cell IFN-γ was analyzed on day 8 post-infection. Results are representative of three-independent experiments involving at least 3 mice per group. Statistical analyses were done using one-way ANOVA with a Tukey’s multiple comparison test, *P<0.05, **P<0.01. Error bars, standard error mean. (TIF) [file ppat.1007872.s007.tif]

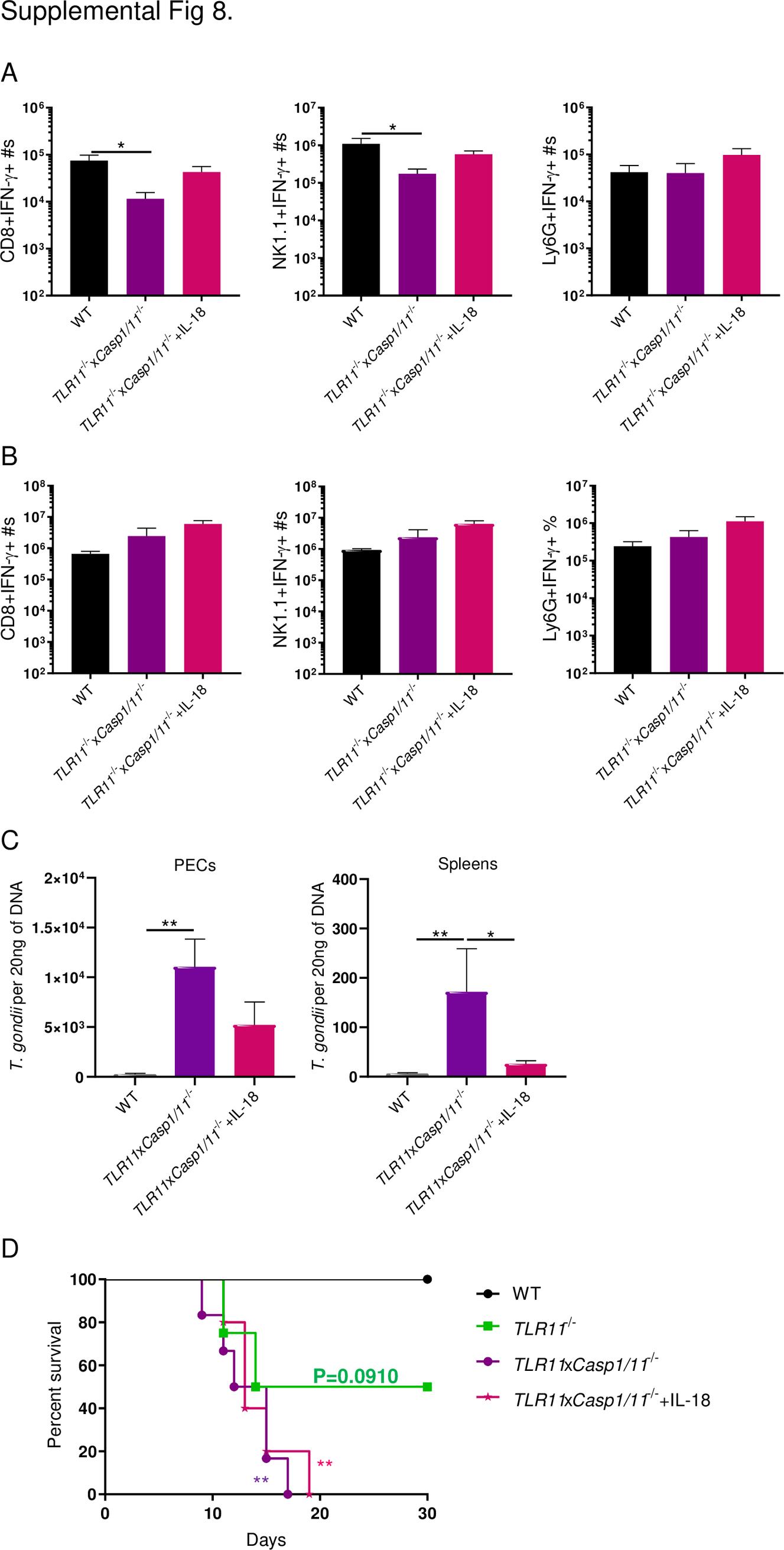

Supplement: S8 Fig — WT, TLR11xCasp1/11-/-, mice were infected i.p. with 20 cysts of T. gondii. TLR11xCasp1/11-/- mice were administered 200 ngs of IL-18 i.p. on days 0, 1, 2, and 3. Absolute quantification of CD8+IFN-γ+, NK1.1+IFN-γ+, and Ly6G+IFN-γ+ cells in the PECs (A) and spleens (B) were analyzed on day 8 following infection. (C) Analysis of T. gondii parasite loads by qPCR from PECs and spleens on day 8 of infection. Results are representative of three-independent experiments involving at least 3 mice per group. (D) Statistical analyses were done using one-way ANOVA with a Tukey’s multiple comparison test, *P<0.05, **P<0.01. Error bars, standard error mean. Survival of WT, Casp1/11-/-, TLR11-/-, TLR11xCasp1/11-/-, and TLR11xCasp1/11-/- mice treated with 200 ngs of IL-18 i.p. on days 0, 1, 2, and 3 post infection. All mice were i.p. infected with 20 cysts of the ME49 strain of T. gondii. Survival curve is representative of three-independent experiments involving at least 5 mice per group. Statistical analyses of survival curve was done using Log-Rank (Mantel Cox) Test, *P<0.05, **P<0.01, ***P<0.001, ****P<0.0001. (TIF) [file ppat.1007872.s008.tif]
